# Cost minimization analysis of treatment with intravenous or subcutaneous trastuzumab in patients with HER2-positive breast cancer in Spain

**DOI:** 10.1007/s12094-017-1684-4

**Published:** 2017-06-02

**Authors:** G. Lopez-Vivanco, J. Salvador, R. Diez, D. López, M. De Salas-Cansado, B. Navarro, J. De la Haba-Rodríguez

**Affiliations:** 1 Hospital de Cruces, Vizcaya, Spain; 2 Hospital Nuestra Señora de Valme, Sevilla, Spain; 3 Hospital de Getafe, Madrid, Spain; 4 Hospital ICO Badalona, Barcelona, Spain; 5 Roche Farma SA, Madrid, Spain; 6 Instituto Maimónides de Investigación Biomédica de Córdoba (IMIBIC), Universidad de Córdoba, Hospital Reina Sofia, Córdoba, Spain

**Keywords:** Trastuzumab, Time and motion, Spain

## Abstract

**Purpose:**

To describe healthcare professional (HCP) and patient time and related costs associated with trastuzumab intravenous infusion (IV) and trastuzumab subcutaneous (SC) formulations in patients with HER2-positive early breast cancer.

**Methods:**

This prospective, observational time, and motion study in three Spanish centers was run as a substudy of the PrefHer trial. We recorded active HCP time for trastuzumab SC and IV-related tasks and calculated HCP time as the mean sum of task times over 154 administrations (80 IV, 74 SC). We calculated mean patient infusion chair time and treatment room time. Staff costs were calculated using fully loaded salary costs based on Spanish salaries (€ 2012).

**Results:**

The transition from trastuzumab IV to SC led to a 50% reduction in active HCP time [27.2 min (95% CI 21.8–32.6) vs. 13.2 min (95% CI 8.9–17.5) per cycle]. Time savings resulted from avoiding IV catheter installation and removal, line flushing, and drug reconstitution. SC administration led to a fivefold reduction (78–85%) in chair time and a fourfold reduction (59–81%) in patient treatment room time, resulting in 24 h free-up time in the total treatment course (18 cycles). Total estimated direct costs were € 29,431.75 and € 28,452.12 for IV and SC, respectively, a saving of € 979.60 over a full treatment course.

**Conclusions:**

Trastuzumab SC provided substantial time savings for HCP and patients, and reduced staff costs vs. trastuzumab IV. Reducing the use of hospital facilities may result in further savings and improved quality of medical care.

**Electronic supplementary material:**

The online version of this article (doi:10.1007/s12094-017-1684-4) contains supplementary material, which is available to authorized users.

## Introduction

Trastuzumab, a humanized monoclonal antibody, is indicated for the treatment of early or metastatic HER2-positive breast cancer and HER2-positive metastatic gastric cancer [1]. In these patients, trastuzumab is administered every 3 weeks for 1 year or until disease progression by intravenous infusion (IV) at a dose calculated according to the patient’s weight [1]. The duration of administration IV is 90 min in the first administration and 30 min for successive administrations [1].

There is also a fixed dose subcutaneous (SC) formulation administered by a single-use injection device (SID). In the enHANced treatment with NeoAdjuvant Herceptin (HannaH) study, conducted in patients with early HER2-positive breast cancer, the SC formulation demonstrated pharmacokinetics, efficacy, and a safety profile comparable to IV, with an administration time of <5 min [2].

Time and motion studies (T&M) are observational studies which examine in detail the times of the operations that make up a process and allow optimization of efficiency and cost reductions. In the health field, T&M studies can analyze the time and resources invested in health processes, which identify steps that can be improved and compare therapeutic strategies in terms of the time spent by healthcare professionals (HCP) and the use of healthcare resources [3]. In oncology, T&M studies have been conducted to evaluate the workload of HCP [4, 5] and analyze the time and resources associated with the preparation and administration of treatments [6–10].

We used a T&M methodology to assess the time and resources associated with the preparation and administration of trastuzumab IV and SC in a subgroup of patients with early HER2-positive breast cancer who participated in the patient preference for subcutaneous (SC) vs. Intravenous (IV) HERceptin (PrefHer) study (NCT01401166) [11, 12] in three Spanish centers [10].

In addition to a shorter administration time, trastuzumab SC does not require a loading dose or calculation based on weight, thereby reducing the preparation time and the likelihood of mistakes. Hopefully, reducing the preparation and administration times will have economic implications and a positive impact on the quality of life.

The aims of this study were: (1) to estimate the HCP time invested in the preparation and administration of trastuzumab IV and SC, patient infusion chair time, patient treatment room time, and patient hospital time according to the route of administration and (2) to estimate the direct costs (costs of HCP, consumables, and drugs) and indirect costs (lost productivity) associated with IV and SC administration.

## Methods

### T&M study methodology

We made a prospective observational study in a subgroup of patients with HER2-positive early breast cancer that participated in the PrefHer study [11] between January and July 2012 in three Spanish centers. All data were collected by external observers during the PrefHer study. The sample size was determined by the number of PrefHer study participants. Each observation consisted of measuring the time required to perform a specific task related to the preparation and administration of trastuzumab.

**Fig. 1:**
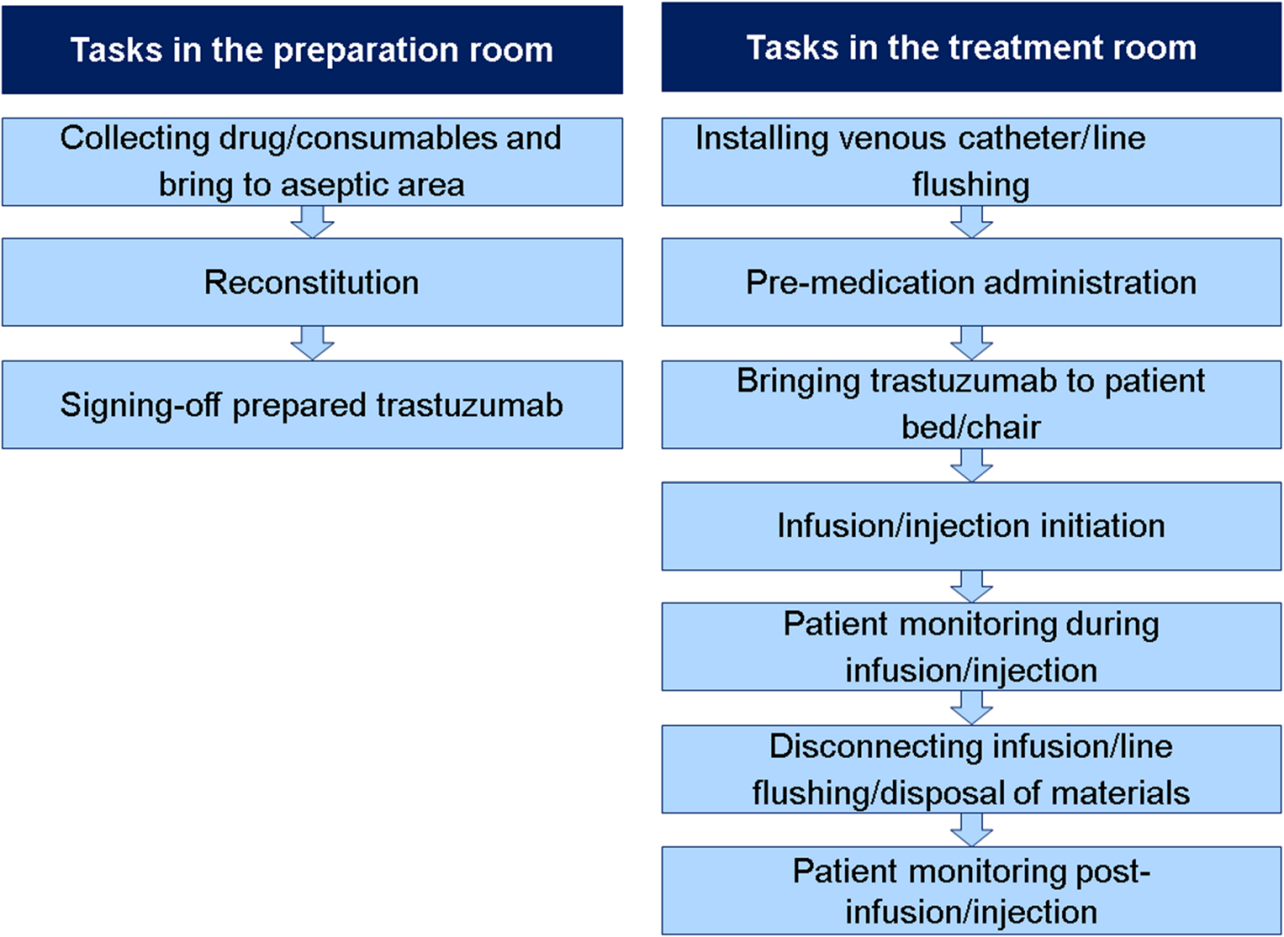
Tasks evaluated in the preparation area and treatment room

To quantify active HCP time, the time actively invested in carrying out the tasks in which differences between the routes of administration had been predicted were observed. Figure 1 shows the tasks observed in the treatment room and the preparation area. All observations were made using a stopwatch.

To quantify patient times, the patient infusion chair time (time between sitting and rising from infusion chair), treatment room time (time between entrance and exit from treatment room), and hospital time (time between entry and exit from the hospital), chair time and treatment room time were determined using a conventional chronometer. Total patient hospital time was determined by patient interview.

All results were calculated for each center and for all centers. For each task, the central trend and the dispersion of data [mean, standard deviation, and 95% confidence intervals (CI)] were calculated using gamma, log-normal, or Weibull continuous probability distributions. Variability between centers was assessed using multilevel models. Since the effect of the centers was not significant, the standard regression models were used. The total time associated with each formulation was calculated by summing the time required for each task. The statistical analysis was made using SAS version 9.2 (SAS Institute Inc., Cary, NY, USA).

### Cost minimization analysis

Direct and indirect costs were calculated. Direct costs included HCP costs for the tasks observed (nurses, pharmacists, and pharmacy technicians), costs of consumables, and drug costs. Indirect costs included the cost of lost productivity (calculated from the patient treatment room and hospital times). All costs were calculated in 2016 euros.

HCP costs were calculated taking into account the gross salary of HCP involved in the activities observed (obtained from the official tariffs of Spanish autonomous communities) and other employment costs (Social Security, training, occupational health, insurance, etc. [13] (Table 1).

**Table 1:** Costs of healthcare workers in Spain (2016 euros)

Professional category	Gross annual salary^a^	Other occupational costs (%)^b^	Total cost/year	Total cost/min
Specialized care nurse	€ 26,206.75	84	€ 48,219.78	€ 0.45
Hospital nursing assistant	€ 18,125.93	84	€ 33,352.48	€ 0.30
Pharmacist	€ 37,985.42	84	€ 69,892.77	€ 0.65

Source: ^a^ Average calculated from gross salaries published in official documents of the Autonomous Community of Aragon (2011) and Generalitat of Valencia (2012) [14, 15] and adjusted according to the retail price index [16]

Source: ^b^ [13]

The costs of consumables were determined using unit costs of public contracts for hospital supply, the General Council of Pharmaceutical Colleges (CGCOF) database, and cost studies (Table 2).

**Table 2:** Costs of consumables in patients treated with trastuzumab IV or trastuzumab SC during a complete cycle Sources: [17–23]

	Unit cost	Ref	Resource use		Costs		
			IV	SC	IV	SC	Dif. IV–SC
Saline solution (ml)	€ 1.25	17	405.7	–	€ 1.014	€ –	€ 1.014
Heparinized solution (3 ml unit)	€ 0.74	18	0.3	–	€ 0.247	€ –	€ 0.247
Injectable water (ml)	€ 1.20	17	7.0	–	€ 0.008	€ –	€ 0.008
Syringe (unit)	€ 0.08	19	3.3	0.3	€ 0.271	€ 0.027	€ 0.244
Needle (unit)	€ 0.52	19	2.7	1.3	€ 1.392	€ 0.696	€ 0.696
Latex gloves (1 pair)	€ 0.03	20	3.7	0.7	€ 0.199	€ 0.036	€ 0.162
Gauze (unit)	€ 0.12	19	5.3	1.3	€ 0.619	€ 0.155	€ 0.464
Dressing (unit)	€ 2.20	19	1.0	0.7	€ 2.204	€ 1.469	€ 0.735
Surgical tape (cm)	€ 0.14	21	13.3	–	€ 0.004	€ –	€ 0.004
Alcohol (ml)	€ 0.35	22	4.0	4.0	€ 0.003	€ 0.003	€ –
Cotton (g)	€ 5.93	17	0.3	–	€ 0.002	€ –	€ 0.002
Catheter or peripheral catheter (unit)	€ 0.88	23	1.3	–	€ 1.171	€ –	€ 1.171
3-way valve (unit)	€ 0.13	23	0.7	–	€ 0.088	€ –	€ 0.088
Transfusion extension tube (unit)	€ 0.12	23	0.3	–	€ 0.041	€ –	€ 0.041
Perfusion pump kit (unit)	€ 1.73	23	0.7	–	€ 1.156	€ –	€ 1.156
Vented spike (unit)	€ 0.13	23	1.00	–	€ 0.132	€ –	€ 0.132
Pump adaptor with spike (unit)	€ 0.13	23	0.7	–	€ 0.088	€ –	€ 0.088
Total					€ 8.64	€ 2.39	€ 6.25

Drug costs were calculated according to reported ex-factory prices of trastuzumab IV 150 mg (€ 596.52) and trastuzumab SC 600 mg (€ 1572.28) [17]. All calculations were performed taking an average patient weight of 66.4 kg (average weight in Spanish women aged 45–54 years [16]) treated with trastuzumab for 18 tri-weekly dosing cycles according to the data sheet guidelines. In line with the standard clinical practice, in patients treated with trastuzumab IV, all vials were considered used. The effect of possible differences between reported and financed prices was assessed in a sensitivity analysis in which discounts of 15% in the ex-factory price of the vial of trastuzumab IV and between 15 and 20% in the ex-factory price of trastuzumab SC were applied. The effect of differences in the weight of patients was evaluated in another sensitivity analysis in which the costs of treatment in patients weighing between 65 and 70 kg were calculated.

Indirect costs were estimated using the human capital method according to the patient treatment room time and hospital times and the average salary in women aged 45–54 years according to the latest update of the Spanish salary survey adjusted according to the retail price index (€ 21,023.57) [24] and the recorded unemployment rate in this group of women in 2015 (21.38%) [16].

## Results

### T&M study results

#### Number of observations

In total, 307 observations were made: 159 in patients with IV administration (80 in the treatment room and 79 in the preparation area) and 148 patients with SC administration (74 in the treatment room and 74 in the preparation area). The number of observations was balanced between centers.

#### Health professional time in the activities observed

**Fig. 2:**
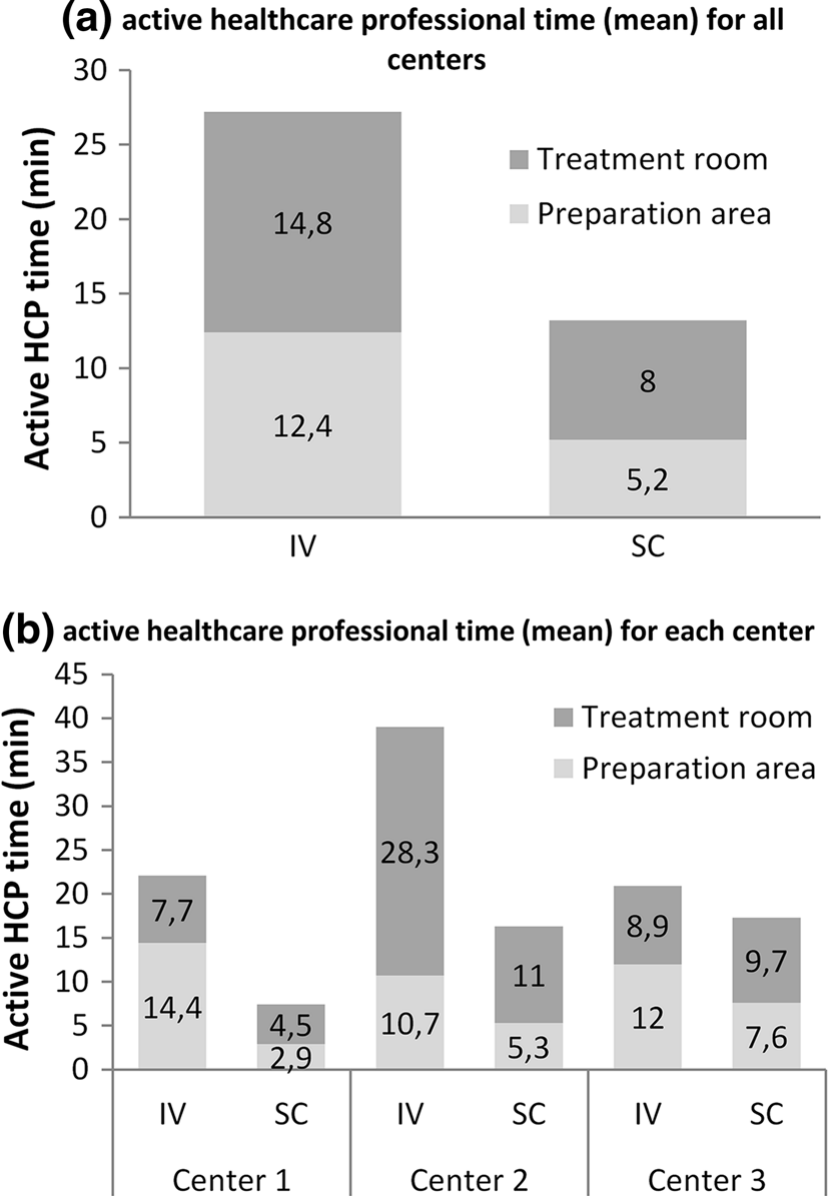
Active healthcare professional time in the tasks observed in the treatment room and the preparation area for all centers (**a**) and for each center (**b**) according to the route of administration

Trastuzumab SC was associated with a reduced active HCP time in all centers, with average times of 13.2 min for trastuzumab SC (95% CI 8.9–17.5) and 27.2 min for trastuzumab IV (95% CI 21.8–32.6), a mean relative reduction of >50%. In all centers, the use of trastuzumab SC resulted in a reduction in the active HCP time compared with trastuzumab IV, with reductions of between 3.6 and 22.7 min in absolute terms and between 17 and 66% in relative terms. Figure 2 shows the times observed in each center and all centers.

A reduced time for all professional categories involved in drug preparation and administration processes was observed. In absolute terms, the greatest reduction was in nursing time (21.8 vs. 11.2 min), but there were also significant differences in the pharmacist time (4.2 vs. 1.2 min) and nursing assistant time (1.1 vs. 0.8 min).

**Fig. 3:**
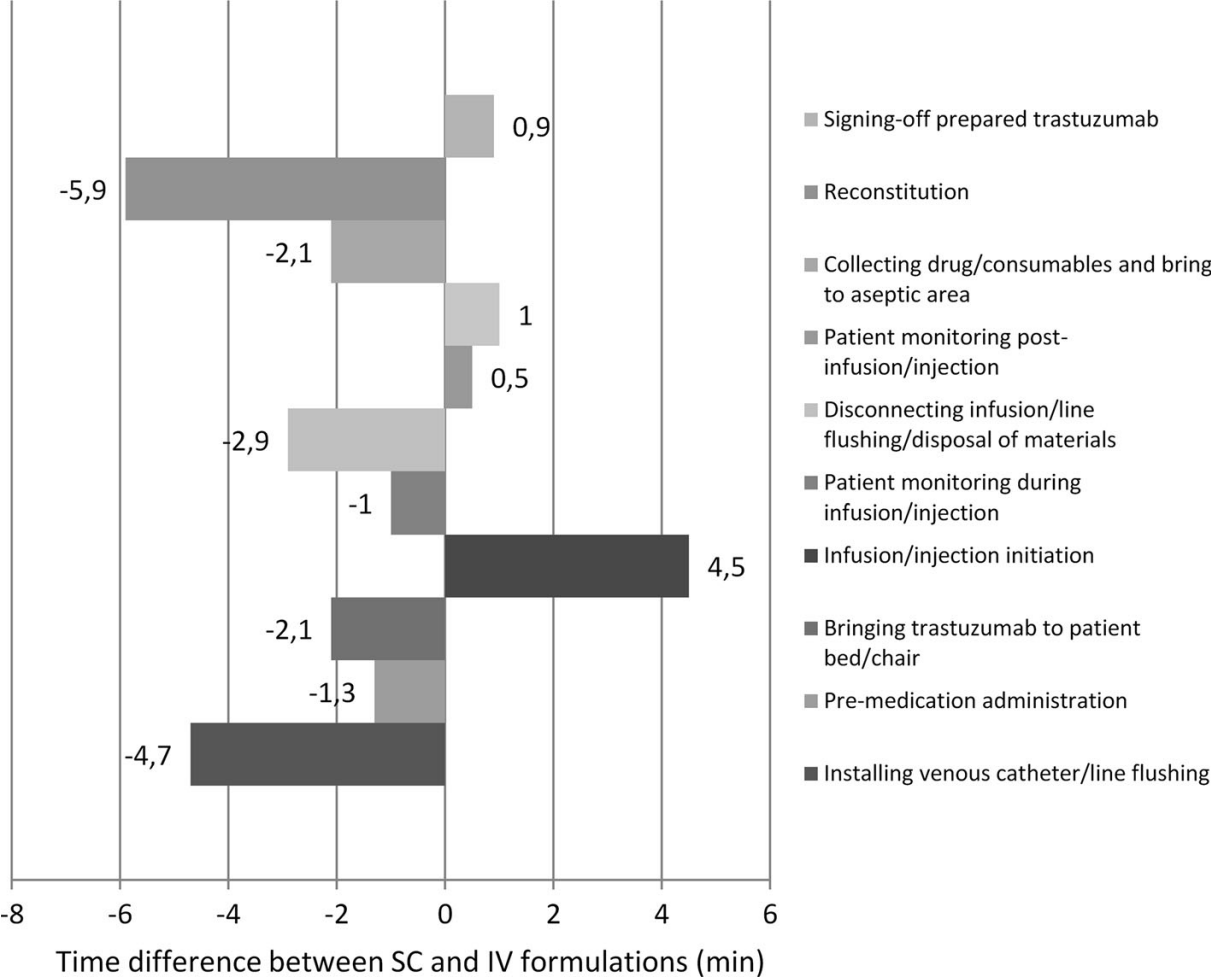
Difference between active times observed between trastuzumab SC and IV (time required for SC minus time required for IV)

In the treatment room, trastuzumab SC resulted in time savings due to the elimination of catheter installation requirements, the administration of premedication, drug transport to the room/chair, and monitoring during the infusion/injection which with the IV formulation required 4.7, 1.3, 2.1, and 1 min, respectively, as well as reducing the downtime and washing or disposal of materials (4.1 vs. 1.2 min). In the preparation area, trastuzumab SC resulted in a reduction in material preparation time (6.0 vs. 3.9 min) and drug reconstitution (5.9 vs. 0 min). Increases in the time required for the start of injection/infusion (1.0 vs. 5.5 min), post-injection/infusion monitoring (0.7 vs. 1.2 min), and authorization of the preparation (0.4 vs. 1.3 min) were compensated for by reductions in the time of other activities. Figure 3 shows the differences between the average times observed for each task.

Extrapolating the results observed to complete treatment cycles, trastuzumab IV would require 8.2 h (95% CI 6.5–9.8) and trastuzumab SC 4 h (95% CI 2.7–5.2). Therefore, in a center that treated ten patients per year, replacing trastuzumab IV by trastuzumab SC would result in an annual reduction in active HCP time of 42 h.

#### Patient chair time, treatment room time, and hospital time

Trastuzumab SC resulted in a reduction of 80% in patient chair time compared with trastuzumab IV (101 vs. 20 min), 45% in treatment room time (120 vs. 30 min), and 44% in hospital time (205 vs. 115 min). Reductions were observed in all centers and there was little variation between centers in the reduction in patient chair time (range 78–85%).

Extrapolation to a population of patients treated for 1 year with 18 cycles of trastuzumab showed that replacing trastuzumab IV by trastuzumab SC in all patients would result in an average saving of 243 h of patient chair time.

### Direct costs

#### Costs of tasks observed

The cost of HCP time invested in the preparation and administration of trastuzumab was € 12.76/cycle of trastuzumab IV and € 6.01/cycle of trastuzumab SC. For a complete 18-cycle treatment, this would result in a cost of € 229.70 for trastuzumab IV and € 108.13 for trastuzumab SC, a difference of € 121.57. Extrapolating these results to a center treating ten patients per year with trastuzumab, the total cost would be € 2297 if all patients received trastuzumab IV and € 1081 if all received trastuzumab SC, an average saving favorable to trastuzumab SC of € 1216 € (−53%).

#### Costs of consumables

The cost of consumables per treatment cycle was € 8.64 for trastuzumab IV and € 2.39 for trastuzumab SC, a difference of € 6.25. For a complete 18-cycle treatment, the cost would be € 155.46 for trastuzumab IV and € 42.95 € for trastuzumab SC, a saving of € 112.52 per patient (Table 2).

#### Drug costs

In the base case (reported ex-factory price and a patient weight of 66.4 kg), the total cost of an 18-cycle treatment would be € 29,046.55 for trastuzumab IV and € 28,301.04 for trastuzumab SC, a difference of € 745.51 in favor of trastuzumab SC.

In the first alternative scenario (discount of 15% for trastuzumab IV and a range of discounts from 15 to 20% for trastuzumab SC), the cost differences between treatments ranged between € 633.68 and € 2048.73. In the second alternative scenario (considering patient weights between 65 and 70 kg), the cost differences between treatments ranged between € 133.08 and € 2320.32. More extreme weights (i.e., patients ≥80 kg) could reach savings greater than € 6500.

### Indirect costs

Estimated indirect costs according to lost productivity measured by patient treatment room time for an 18-cycle treatment were € 203.78 (loss of 4.5 working days) for trastuzumab IV and € 50.94 (loss of 1.1 working days) for trastuzumab SC. Estimated indirect costs according to lost productivity measured by total hospital time for an 18-cycle treatment were € 348.12 (loss of 7.7 working days) for trastuzumab IV and € 195.29 (loss of 4.3 working days) for trastuzumab SC. Therefore, regardless of the approach used to determine lost productivity, trastuzumab SC resulted in a reduction in indirect costs of € 152.83 per patient compared with trastuzumab IV.

### Total costs

Direct costs were € 29,431.72 for trastuzumab IV and € 28,452.12 for trastuzumab SC, a net difference in favor of trastuzumab SC of € 979.60. When indirect costs were added, replacement of trastuzumab IV by trastuzumab SC for a full 18-cycle treatment would save € 1132.43 (Table 3).

**Table 3:** Total costs in patients treated with trastuzumab IV or trastuzumab SC

	IV	SC	Difference
Direct costs	€ 29,431.72	€ 28,452.12	€ 979.60
Healthcare professional costs	€ 229.70	€ 108.13	€ 121.57
Consumable costs	€ 155.46	€ 42.95	€ 112.52
Drug costs	€ 29,046.55	€ 28,301.04	€ 745.51
Indirect costs	€ 203.78	€ 50.94	€ 152.83
Total costs	€ 29,635.49	€ 28,503.06	€ 1132.43

## Discussion

This study describes active HCP time invested in the preparation and administration of trastuzumab and shows that replacement of trastuzumab IV by trastuzumab SC would reduce active HCP time by 50%. While HCP could dedicate part of the non-active time to other activities, the requirement for monitoring during and after infusion/injection would limit the other activities that HCP could perform in parallel. In addition, greater available HCP time could result in improvements in the quality of care, with more time free for monitoring or patient information.

Compared with centers in other countries in the T&M study in which an SID device was used, the saving in active HCP time observed in the Spanish centers (50%) was similar to that observed in Canada and Russia (48%) and much higher than in France (36%), Denmark (31%), and Switzerland (15%) [10]. In addition, the relative difference in patient chair time was greater in Spain (80%) than in the other countries (66–79%) [10], suggesting that the consequences of replacing trastuzumab IV by trastuzumab SC would be greater in Spain than in comparable countries.

The results show that replacing trastuzumab IV by trastuzumab SC would result in a saving of € 121.6 per patient in active HCP time for a full 18-cycle treatment, consistent with studies conducted in Belgium, France, New Zealand, the United Kingdom, Germany, and Venezuela [25–30].

We only measured mean chair time without defining the associated direct costs. However, reduced chair time would reduce the overall use of health facilities which, in the long term, could also result in cost savings. Reductions in chair time and treatment room time could allow the treatment of the same number of patients with fewer resources or more patients with the same resources. Even without considering that reducing chair time could allow more patients to be treated, according to the time-opportunity concept, the results suggest that optimization of HCP times would lead to improvements in care quality, i.e., the same number of patients treated but with improved care quality.

Likewise, as trastuzumab SC uses a fixed dose, this would result in savings in drug costs of € 745.5 per patient/complete treatment.

The reduction in patient treatment room time resulted in a reduction in indirect costs due to lost productivity of € 152.83 per 18-cycle treatment, a conservative estimate which only considered lost productivity between entering and leaving the hospital and not travel to the hospital (especially relevant in rural areas) or the time lost by accompanying persons. In addition to the economic implications, the reductions in time associated with trastuzumab SC could have implications for the quality of life. In fact, in the PrefHer study, the time saved by the patient was the reason for preferring SC over IV treatment [12]. Therefore, beyond an estimate of costs from the social perspective, according to the preferences expressed, the major beneficiaries of trastuzumab could be the patients themselves.

The main limitation of the study is that T&M was not designed to determine the significance of the differences observed between the IV and SC formulations. However, the lack of overlap of the 95% CI for active HCP time (IV: 21.8–32.6 min vs. SC: 8.9–17.5 min) and patient chair time (IV: 91.7–112.1 min vs. SC: 16.9–24.6 min) suggests differences in the time and costs calculated for the two groups.

Another possible limitation is that the study was conducted in a small number of centers in the context of a clinical trial and there could be differences between the times observed in the study and in real clinical practice. However, replacement of trastuzumab IV by trastuzumab SC resulted in simplification of the process of preparation and administration and a reduction in all associated times for all professional categories in all study centers. Moreover, greater experience in the use and handling of the SID will significantly affect administration and chair times, suggesting that, in clinical practice, further reductions in the administration time will occur.

## Conclusion

In conclusion, the replacement of trastuzumab IV by trastuzumab SC would reduce active HCP time, patient chair time, and patient hospital time, thereby improving patients’ quality of life. In the Spanish health context, these reductions in time would result in economic savings, more efficient resource use and improved quality of care. Trastuzumab SC use would also reduce consumable and drug costs. A full cycle of trastuzumab SC would result in a saving of € 979.60 in direct costs. These savings could be greater if patients could avoid going to hospital, or at least increasing the use of rapid administration, further optimizing the healthcare burden in the hospital settings. Throughout Spain, between 4000 and 5000 women would be eligible for treatment with trastuzumab for early breast cancer, resulting in a saving for the health system of more than four million euros. The widespread use of trastuzumab SC would also result in a reduction in indirect costs due to less work productivity lost. These clinical and economic aspects show that trastuzumab SC results in benefits for patients, HCP, and society in general, and is being adopted as the standard treatment for HER2 + breast cancer.

## Electronic supplementary material

Below is the link to the electronic supplementary material.

Supplementary material 1 (JPEG 153 kb)

Supplementary material 2 (DOCX 70 kb)
