# Supplementary figures and images for: Cost minimization analysis of treatment with intravenous or subcutaneous trastuzumab in patients with HER2-positive breast cancer in Spain
Source: Clin Transl Oncol. 2017 Jun 2;19(12):1454–61. doi: 10.1007/s12094-017-1684-4 (PMC5700215; doi:10.1007/s12094-017-1684-4)

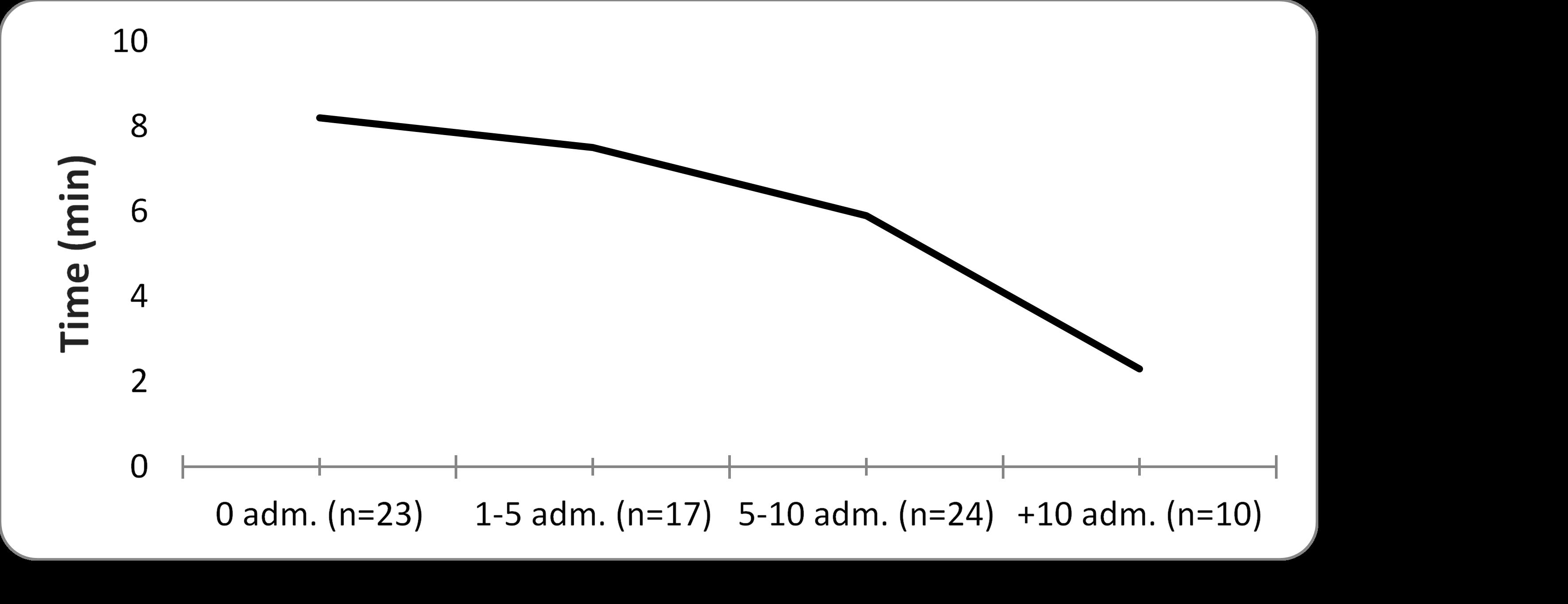

Supplement: Supplementary file 1 — Supplementary material 1 (JPEG 153 kb) [file 12094_2017_1684_MOESM1_ESM.jpg]
