# Supplementary material for: Cost minimization analysis of treatment with intravenous or subcutaneous trastuzumab in patients with HER2-positive breast cancer in Spain
Source: Clin Transl Oncol. 2017 Jun 2;19(12):1454–61. doi: 10.1007/s12094-017-1684-4 (PMC5700215; doi:10.1007/s12094-017-1684-4)
Supplement: Supplementary file 2 — Supplementary material 2 (DOCX 70 kb) [file 12094_2017_1684_MOESM2_ESM.docx]

**SUPPLEMENTARY MATERIAL**

*Analysis of covariance*

Analysis of covariance was made to assess the influence of various factors on the time required to perform specific tasks. We evaluated: 1) the effect of peripheral or permanent access on the time required for the installation and washing of the venous catheter, the transport of trastuzumab, the start of infusion, disconnection and the disposal of materials and patient chair time; 2) the effect of using premedication on the time required for the start of infusion, transport of trastuzumab, administration and patient chair time; 3) the effect of the first administration versus successive administrations on the time required for starting the infusion, monitoring during the infusion, monitoring after the infusion, and patient chair time; 4) the effect of the level of experience in SID administration (never, 1-5 times, 5-10 times, +10 times) on the time required for the preparation of trastuzumab, administration of the injection, disposal of the SID, post-injection monitoring and patient chair time.

Premedication had a statistically significant effect on the time required for the start of the infusion (1.23 vs. 0.49 min; p <0.0001) and patient chair time (113.15 vs. 67.71 min; p <0.0001).

The level of experience of HCP significantly affected SC injection time (p <0.01) (Fig. 5) and patient chair time (p <0.01): as experience increased, a reduction in the time required for the completion of all tasks relating directly to trastuzumab SC injection was observed.

In processes related to trastuzumab IV, the availability of permanent access (versus peripheral access) and the first administration (versus successive administrations) did not significantly affect the time required for the tasks evaluated.

**Fig. S1 Evolution of the time required for the injection of trastuzumab SC according to the number of previous administrations.**
